# Quantitative Profiling of the Effects of Vanoxerine on Human Cardiac Ion Channels and its Application to Cardiac Risk

**DOI:** 10.1038/srep17623

**Published:** 2015-11-30

**Authors:** Carlos A. Obejero-Paz, Andrew Bruening-Wright, James Kramer, Peter Hawryluk, Milos Tatalovic, Howard C. Dittrich, Arthur M. Brown

**Affiliations:** 1 ChanTest Corporation, a Charles River Company, Discovery Services, 14656 Neo Parkway, Cleveland, OH 44128, USA; 2 Laguna Pharmaceuticals, 4225 Executive Square, Suite 960, La Jolla, CA 92037, USA

## Abstract

Vanoxerine has been in clinical trials for Parkinsonism, depression and cocaine addiction but lacked efficacy. Although a potent blocker of hERG, it produced no serious adverse events. We attributed the unexpected result to offsetting Multiple Ion Channel Effects (MICE). Vanoxerine’s effects were strongly frequency-dependent and we repositioned it for treatment of atrial fibrillation and flutter. Vanoxerine terminated AF/AFL in an animal model and a dose-ranging clinical trial. Reversion to normal rhythm was associated with QT prolongation yet absent proarrhythmia markers for Torsade de Pointes (TdP). To understand the QT/TdP discordance, we used quantitative profiling and compared vanoxerine with dofetilide, a selective hERG-blocking torsadogen used for intractable AF, verapamil, a non-torsadogenic MICE comparator and bepridil, a torsadogenic MICE comparator. At clinically relevant concentrations, verapamil blocked hCav1.2 and hERG, as did vanoxerine and bepridil both of which also blocked hNav1.5. In acute experiments and simulations, dofetilide produced early after depolarizations (EADs) and arrhythmias, whereas verapamil, vanoxerine and bepridil produced no proarrhythmia markers. Of the MICE drugs only bepridil inhibited hERG trafficking following overnight exposure. The results are consistent with the emphasis on MICE of the CiPA assay. Additionally we propose that trafficking inhibition of hERG be added to CiPA.

Vanoxerine, 1-[2-[*bis*(4-fluorophenyl)methoxy]ethyl]-4-[3-phenylpropyl] piperazine dihydrochloride (synonym GBR-12909), is a potent, highly selective dopamine transporter antagonist developed initially for treatment of Parkinson’s disease and depression. Vanoxerine was safe in a clinical tolerance study^1^, a Phase 1 trial^2^ and recently, the COR-ART clinical trial to evaluate the effect of the drug on the conversion of AF/AFL to normal sinus rhythm (NSR)^3^.

During a preclinical work-up for use in cocaine addiction, Brown and colleagues found that vanoxerine was a potent hERG blocker^4^. This result was at odds with the absence of serious adverse cardiac events in human studies as predicated by the S7B and E14 guidances^5^,^6^ based on the causative hypothesis of hERG block, QT prolongation and Torsade de Pointes (TdP). To explain the discordance we proposed a more general hypothesis invoking multiple ion channel effects (MICE) in which block of inward sodium and calcium currents offset block of the outward hERG potassium currents as is the case for verapamil and amiodarone^4^,^7^. As confirmation, Lacerda *et al.* (2010) showed that vanoxerine blocked human cardiac sodium (hNav 1.5) channel currents and guinea pig cardiac calcium currents in addition to blocking hERG channel currents and prolonged action potential durations (APDs) mildly in canine cardiac myocytes and Purkinje fibers. By contrast, dofetilide a potent, selective hERG blocker used to treat AF/AFL prolonged APD^4^ and QT and produced Torsade de Pointes^7^,^8^. Vanoxerine’s block of Cav 1.2 and Nav 1.5 in particular were more potent at faster rates^4^ and led to the suggestion that vanoxerine might be effective in terminating AF/AFL and restoring NSR without being proarrhythmic. If correct, this would be very important since AF/AFL is prevalent, serious, increasing in frequency and without satisfactory medical treatment. We tested vanoxerine’s antiarrhythmic potential in a sterile pericarditis canine model of AF/AFL and found that vanoxerine administered orally or intravenously terminated AF/AFL and restored NSR without producing arrhythmias^9^. In an extension of this study we found that vanoxerine prevented re-induction of AF/AFL^10^. More recently and most importantly, similar results were obtained in a multicenter, randomized, double-blind, placebo-controlled, ascending oral dose, clinical trial^3^.

In our earlier paper, we used different voltage protocols for hERG and hNav 1.5 channel currents, measured calcium currents in guinea pig cardiomyocytes with yet another protocol and measured cardiac action potentials in canines^4^. Vanoxerine’s effects while consistent with the MICE hypothesis^8^,^11^ were interpreted qualitatively. Here we used a uniform approach to provide a quantitative description of vanoxerine’s MICE profile in order to understand the drug’s actions on experimental and simulated human ventricular action potentials. We achieved this by: 1) using similar step-ramp, cardiac-like, voltage protocols for hERG, hNav 1.5 and hCav 1.2 channels each of which was expressed heterologously in HEK 293 or CHO cell lines and measuring concentration-responses (CRs) of vanoxerine block in the steady state using manual patch clamp; 2) measuring the drug’s effects on human induced pluripotent stem cell (iPSC)-derived cardiomyocyte action potentials (SC-CMAPs); and 3) comparing the experimental action potentials with simulated action potentials using the O’Hara-Rudy model^12^ of the human left ventricular action potential (hVAP). For the latter, conductances were modified according to the experimentally measured CRs of each channel current. In addition to vanoxerine we profiled one non-torsadogenic MICE comparator verapamil, one torsadogenic MICE comparator bepridil and the selective hERG blocker dofetilide. We showed that dofetilide prolonged SC-CMAPs experimentally and in hVAP simulations and produced proarrhythmia markers including EADs and arrhythmias at *in vitro* concentrations comparable to clinical exposures. Verapamil blocked hERG and hCav 1.2 *in vitro* at clinically relevant concentrations and showed no proarrhythmia markers in either experiments or simulations. Unlike verapamil which is ineffective in AF/AFL^13^, vanoxerine also blocked peak and late hNav 1.5 currents. As expected vanoxerine despite APD prolongation produced no proarrhythmia markers. Unexpectedly bepridil showed no proarrhythmia markers in either experiment or simulation. While bepridil and vanoxerine had comparable MICE profiles only bepridil reduced surface expression of wild type hERG following overnight exposure. Neither verapamil nor dofetilide inhibited trafficking of wild type hERG.

In summary, we have shown that MICE drugs like verapamil and vanoxerine need not be torsadogenic despite hERG block and that the torsadogenicity of the MICE comparator bepridil resides in its inhibition of hERG trafficking. The conclusion supports the new regulatory approach embodied by the Comprehensive *in vitro* Proarrhythmia Assay (CiPA) in which cardiac safety is assessed using MICE and proarrhythmic liability rather than hERG block or APD/QT prolongation^14^.

## Results

### Voltage clamp experiments

**Figure 1:**
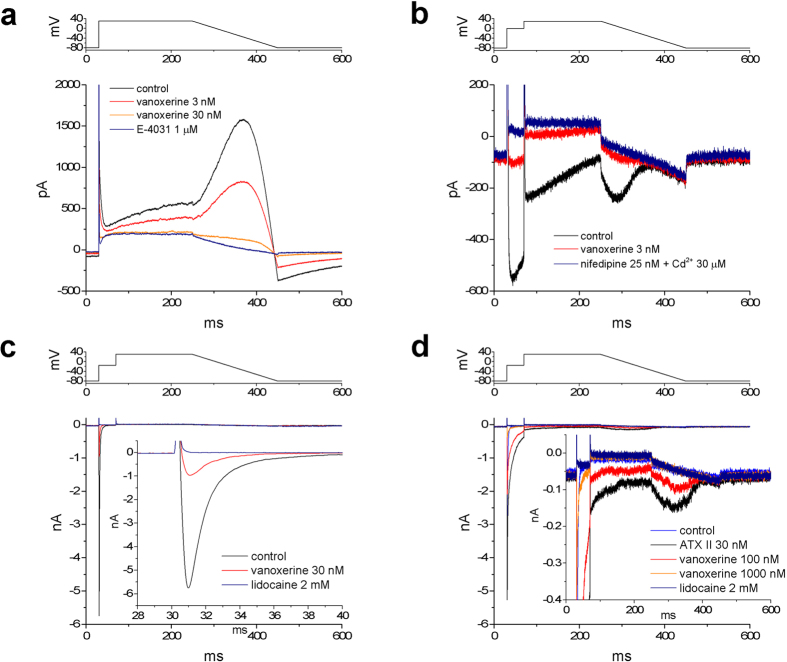
(**a**) Top: Step-ramp voltage protocol applied at 1 Hz used to measure block of hERG channel currents produced by vanoxerine (channels expressed in HEK293 cells at room temperature RT, ~22 °C). Bottom: superimposed currents recorded in control, 3 and 30 nM vanoxerine and 1 μM E-4031. (**b**) Top: protocol at 1 Hz used to measure block of hCav1.2 channel currents (CHO cells, RT ~ 22 °C). The initial step in the step-ramp protocol is to zero mV where the leak current is minimal and the peak current is measured. Bottom: experiment showing block produced by 30 nM vanoxerine and 25 nM nifedipine +30 μM Cd^2+^. Currents were recorded using perforated patch clamp with amphotericin B. (**c**) Top: voltage protocol at 1 Hz used to evaluate block of peak hNav 1.5 channel currents (HEK 293 cells, RT ~ 22 °C). The initial step in the step-ramp protocol is to −15 mV where open probability is approximately maximal and the driving force for Na^+^ is reduced providing better voltage control. Inset: blocking effects of 30 nM vanoxerine and 2 mM lidocaine on an expanded time scale. (**d**) Top: voltage protocol at 1 Hz used to measure block of ATX II- activated late hNav1.5 channel currents (ATX at 30 nM). Late currents were measured at their maxima during the ramp. Inset: block by vanoxerine at 100 and 1000 nM and lidocaine at 2 mM on an expanded current scale.

The present *in vitro* experiments used similar cardiac action potential-like step-ramps delivered at 1 Hz from holding potentials of −80 mV for all three heterologously expressed channel currents. Drug effects were corrected for rundown as described in Methods. Figure 1 shows examples of the four currents studied using the step-ramp voltage protocol. The time courses and CRs of these experiments are shown in Supplementary Figures 1 and 2 respectively.

Figure 1a shows the voltage protocol and associated hERG channel currents before and after acute exposure to 3 and 30 nM vanoxerine. Exposure to 1 μM E-4031 was used to define the leak current. The CR of peak currents recorded during the ramp was fit to a single binding isotherm with an IC50 of 9.3 ± 1.3 nM (95% CI: 7.2–11.8) and a Hill coefficient of 1.11 ± 0.15 (95% CI: 0.84–1.48) (Supplementary Figure 2). This value is one order of magnitude higher than the 0.8 nM value reported previously using 2 s pulses to +20 mV elicited at 0.1 Hz, a voltage protocol that favors the inactivated state more strongly thereby enhancing potency^4^.

Figure 1b shows hCav 1.2 currents in control and after exposure to 30 nM vanoxerine. The leak current was obtained after blocking the channels with 25 μM nifedipine and 30 μM Cd^2+^. The CR of peak currents recorded at 0 mV where leakage current is minimal was fit to a single binding isotherm with an IC50 of 16.2 ± 2.5 nM (95% CI: 11.0–22.0) and a Hill coefficient of 0.63 ± 0.10 (95% CI: 0.44–0.85) (Supplementary Figure 2)+. These values are significantly more potent than the 320 nM value reported by Lacerda *et al.* (2010) using guinea pig ventricular myocytes paced at low frequency (0.05 Hz).

Figure 1c shows the effect of 100 nM vanoxerine on peak hNav 1.5 currents recorded at −15 mV and the effect of 2 mM lidocaine to define the leak current. The effect on peak current was parameterized with a single binding isotherm having an IC50 of 34.6 ± 5.5 nM (95% CI: 23.4–48.8) and a Hill coefficient of 0.97 ± 0.20 (95% CI: 0.57–1.48) (Supplementary Figure 2). This value is significantly lower than the 830 nM value reported by Lacerda *et al.* (2010)^4^, using a protocol that maximized tonic block (hyperpolarizing prepulse to −120 mV, 0.1 Hz stimulation).

Figure 1d shows the effect of 100 and 1000 nM vanoxerine and 2 mM lidocaine on late hNav 1.5 currents activated by 30 nM ATX II. Late hNav 1.5 was measured during the ramp where currents are larger due to the increased driving force for Na^+^. The more hyperpolarized region of the ramp where channels are closed was used to define the leak current of the entire ramp by fitting a straight line extrapolated to the start of the ramp. The effect of vanoxerine was parameterized with a single binding isotherm having an IC50 of 85.2 ± 6.3 nM (95% CI: 72.9–99.9) and a Hill coefficient of 1.62 ± 0.17 (95% CI: 1.30–2.09) (Supplementary Figure 2). The measured IC50s and Hill coefficients for the four currents studied are shown in Table 1. The Table also includes the parameters for block of hKir 2.1 and hKvLQT1/minK channel currents from Lacerda *et al.*, (2010)^4^.

**Figure 2:**
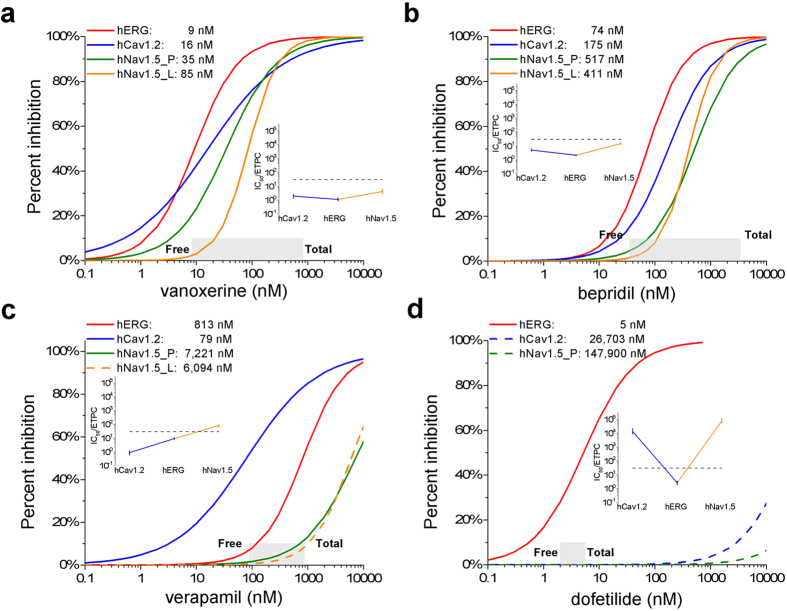
(**a**) CRs for block of hERG, hCav1.2, and peak and late hNav1.5 channel currents by vanoxerine; (**b**) CRs for block of hERG, hCav1.2, peak and late hNav1.5 by bepridil; (**c**) CRs for block of hERG, hCav1.2 and hNav1.5 by verapamil; (**d**) CRs for block of hERG, hCav1.2 and hNav1.5 by dofetilide. Continuous lines indicate data obtained using the step-ramp protocol and are new. Dashed lines in the dofetilide panel indicate CRs for hCav1.2 and hNav1.5 block from Kramer *et al.* (2013). The dashed line in the verapamil panel is the CRs for late hNav1.5 block measured with a pulse protocol. Measured IC50 values are shown. The gray region in the vanoxerine CRs extends from the peak clinical Cmax of 831 nM^1^ to the calculated free concentration assuming 99% plasma protein binding^4^. The respective gray areas in the bepridil, dofetilide and verapamil CRs were calculated from data in Kramer *et al.* (2013). Insets show SaVety™ plots. Blue segments indicate the SM difference between block of hCav1.2 and hERG. Orange segments indicate the SM difference between block of peak hNav1.5 and hERG. SM for hERG is at the intercept of hCav 1.2 and hNav 1.5 segments. Vertical lines indicate the 95% confidence intervals of the ETPC indexes. The dashed line is at a margin of 30 which has been used to identify torsadogenicity due to hERG block alone^14^.

To compare MICE effects, we measured block of hERG, hCav 1.2 and hNav 1.5 channel currents using concentrations related to drug exposure levels. Figure 2 shows the CRs of vanoxerine and the comparators bepridil, dofetilide and verapamil for hERG, hCav 1.2 and peak and late hNav 1.5 currents. Continuous lines are binding isotherms from data measured using step-ramp protocols whereas dashed lines are binding isotherms measured using step pulses. The measured IC50s and Hill coefficients for the four currents and four drugs are compiled in Table 1. The number of experiments associated with the data points that were used for fitting the CRs is shown in Supplementary Figure 2.

The gray region above the x axis in Fig. 2a illustrates the range of concentrations between the peak clinical Cmax of total vanoxerine at 831 nM^1^ and a free value of 8 nM assuming 99% protein binding as reported by Lacerda *et al.* (2010)^4^. At 8 nM all three peak channel currents are clearly reduced along with a smaller reduction in late hNav 1.5. The inset shows the safety margins (SMs) calculated as IC50/ETPC (effective therapeutic plasma concentration) and the relationship between Cav1.2 (blue), Nav1.5 (yellow) and hERG at the vertex of these SaVety^TM^ or V plots^8^. We reported previously that torsadogenic drugs have strong Vs with large differences between the SMs of hERG, hCav 1.2 and hNav 1.5. These differences reflect the absence of offsetting effects from MICE e.g., Fig. 2d dofetilide. For drugs where hERG block is offset by hCav 1.2 and/or hNav 1.5 block the V plots are weak (Fig. 2a, vanoxerine, Fig. 2b, bepridil or inverted Fig. 2c, verapamil) even in the presence of strong hERG block.

The dashed horizontal line in the insets indicates a SM of 30 for hERG block. Drugs with SMs <30 were assigned odds ratios for ventricular arrhythmias up to four times greater than drugs above the line^7^,^15^. While this is true for selective hERG blockers it need not be the case for MICE drugs that block offsetting ion channel currents.

At their free plasma levels verapamil and dofetilide behave mainly as calcium and hERG blockers respectively. Bepridil on the other hand blocks both hERG and Cav1.2 currents. Although calcium block is significantly less potent (Table 1), the overall SaVety™ profile for bepridil is comparable to vanoxerine and in both cases block of hERG, Cav1.2 and Nav1.5 occur at clinical exposure levels.

### Effect of vanoxerine, bepridil, dofetilide and verapamil on human SC-CMAPs

To evaluate drug effects on cardiac excitability we used current clamp patch clamp experiments on SC-CMAPs and simulations based on the O’Hara-Rudy model of hVAPs^12^. Experiments and simulations were performed at and above exposure levels to test for proarrhythmic markers namely EADs, ectopic beats and/or tachycardias.

**Figure 3:**
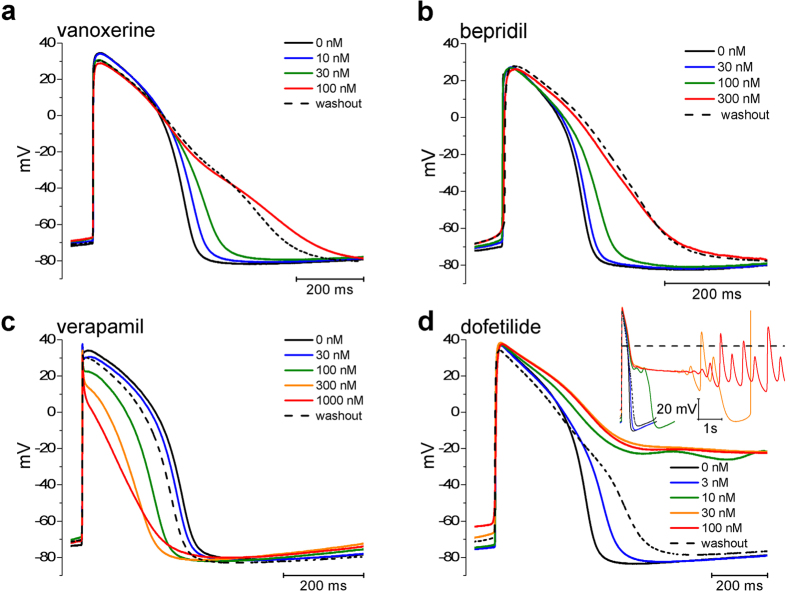
Effect of vanoxerine, bepridil, dofetilide and verapamil on spontaneously beating SC-CMAPs. Spontaneous frequency was ~1 Hz. Note the reduction in action potential amplitude in vanoxerine, bepridil and verapamil and the depolarization of the maximum diastolic potential membrane potential in dofetilide.

Figure 3 shows the effects of vanoxerine and comparators on action potentials measured after at least 5 min exposure to different concentrations and after washout. The time course of changes in membrane potential and APD30, 60 and 90 are shown in Supplementary Figure 3. We restricted our measurements to beat rates between 0.5 and 1.5 Hz.

Figure 3a shows that vanoxerine, as expected from its MICE, impacted three major AP parameters APD90, Vmax and Phase 2 (plateau) amplitude reflecting block of hERG, hNav1.5, and hCav1.2 respectively. Vanoxerine decreased the plateau potential and prolonged the APD60 and 90. It reduced Vmax by 25% (p = 0.062) at concentrations where hNav 1.5 block is significant and maximum diastolic potential is unaffected. No proarrhythmic events were recorded at free concentrations of vanoxerine as large as 100 nM (Supplementary Table S1). An exception occurred in an outlier cell with low beat frequency (0.4 Hz) and an unusually long APD90 for which we observed EADs at 100 nM. By contrast dofetilide at 10–100 nM produced EADs and polymorphic VT-like waveforms (Fig. 3d inset). Supplementary Figure 3 shows that for dofetilide the pattern of APs is replaced by runs of ventricular tachycardia (VT) at the higher concentrations (underscored in panel d). Prior to the arrhythmias dofetilide at 3 nM prolonged APDs 60 and 90 significantly whereas APD30 was slightly prolonged. On average dofetilide at 3 nM increased APD90 by 24% within 5 min of exposure and at 10 nM 90% before cells showed EADs and VT. Unlike dofetilide, bepridil decreased the early plateau and prolonged the duration of the action potential without showing any proarrhythmic markers. Vanoxerine behaved similarly (compare Supplementary tables S1 and S2). Verapamil reduced the plateau and at the highest concentration slightly prolonged the late APD. There were no proarrhythmic events. The effects of the three comparators on the action potential parameters are summarized in Supplementary Tables S2, S3 and S4.

### *In silico* studies

We used the O’Hara-Rudy model to simulate action potentials using data obtained from heterologously-expressed human cardiac ion channel currents. We used the conductance block approach based on measured IC50 and Hill coefficients to scale the maximum conductances in the model equations for hERG, hNav 1.5 and hCav 1.2 currents. A similar approach was used by Mirams *et al.*^16^,^17^. We added blocking potencies for IKs, Kir2.1 and NCX currents when data were available. We used clinically relevant free concentrations from zero to 100 nM in 10 nM increments for vanoxerine, 5 nM increments for dofetilide, from zero to 1000 nM in 100 nM increments for verapamil and from zero to 300 nM in 30 nM increments for bepridil.

**Figure 4:**
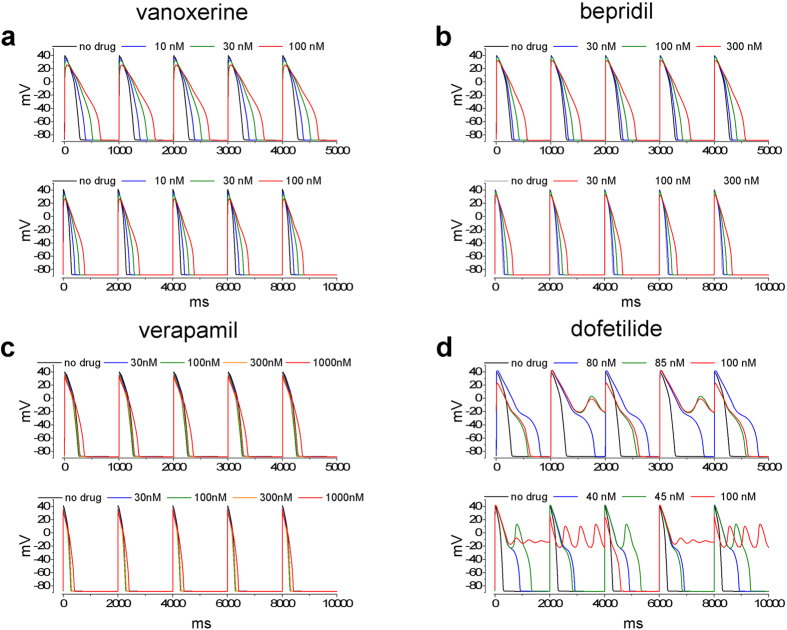
Steady state simulations of action potentials at drug concentrations indicated in the Figure. Indicated simulations were paced at 1 Hz (top panel) and 0.5 Hz (Bottom panel). Records in dofetilide are control (no drug), last record in the series without EADs (blue line, 80 nM at 1 Hz and 40 nM at 0.5 Hz), first record in the series showing EADs (green line, 80 nM at 1 Hz, 45 nM at 0.5 Hz) and maximal tested concentration (red line, 100 nM). Records in verapamil are simulations at the same concentrations used in the stem cell action potentials.

Figure 4 shows simulated potentials at 1 and 2 s periods (top and bottom panels, respectively). Supplementary Figure 4 shows the functional relationships between concentration, frequency and APD90 for vanoxerine and its comparators. From inspection of Fig. 4 for dofetilide EADs appear followed by triggered ectopic beats. The threshold of 40 nM at 2 s periods approximates the 10 nM threshold for triggered activity observed experimentally in SC-CMAPs (Supplementary Table S4). By contrast, vanoxerine, bepridil and verapamil were free of EADs and triggered arrhythmias over the same range of frequencies. The simulation in Fig. 4a shows the reduction in plateau due to vanoxerine block of hCav 1.2 and hNav 1.5 and prolongation of APD90 due to hERG block observed experimentally. For verapamil and bepridil, the simulations resembled the experimental observations with decreased plateau potential and changes in APD90. Supplementary Figure 5 shows single simulated action potentials which compare favorably to the single experimental action potentials of text Fig. 3.

Bepridil prolonged hSC-CMAPs and reduced plateau amplitudes consistent with its block of hERG and Cav 1.2 currents. At 300 nM (8.6 times the free plasma concentration) bepridil prolonged experimental APD90 by 91% and prolonged simulated APD90s at 1 and 0.5 Hz by 106% and 123% respectively (Supplementary Figure 4 and Supplementary Table S2). We anticipated that bepridil being a torsadogenic MICE drug would manifest proarrhythmia markers such as EADs and ectopic beats but such was not the case either experimentally or in simulations. We therefore considered whether it’s torsadogenicity was related to longer exposures since in the clinic TdP occurred after 38 ± 30 days delay following exposure^18^.

### HERG trafficking studies

We developed the hERG-Lite assay based on the chemiluminescence of labeled hERG channels expressed at the cell surface of HEK 293 cells^19^ to determine whether torsadogenic drugs that do not directly block hERG act indirectly by inhibiting hERG trafficking after a delay related to HERG turnover. These were called Class A drugs and were exemplified by pentamidine, arsenic trioxide and geldanamycin. Verapamil and vanoxerine (GBR12909) which produce direct block of hERG current do not inhibit trafficking of wild type hERG after overnight exposure. In fact, direct block by these drugs is associated with rescue and increased expression of a single mutant hERG channel that otherwise is trafficking defective. These drugs were described as Class B drugs. Bepridil belongs to a third class of direct hERG blockers that significantly inhibit trafficking of wild type hERG after overnight exposure. These drugs were described as Class C.

**Figure 5:**
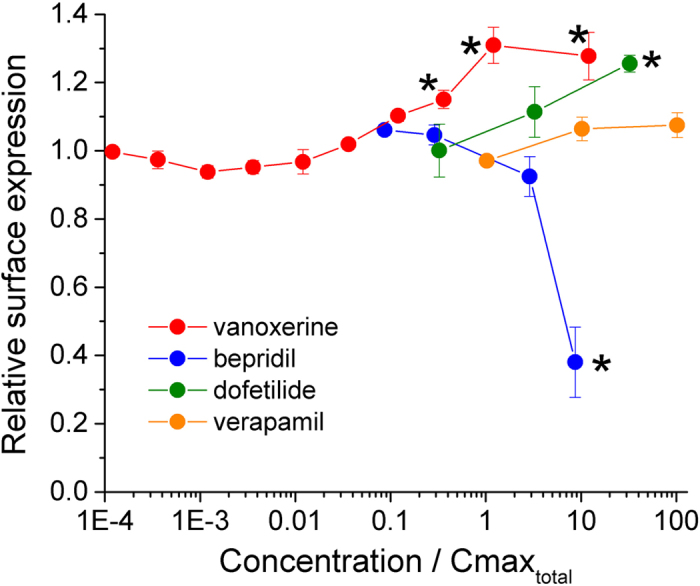
Change in hERG surface expression relative to vehicle control for vanoxerine, bepridil, verapamil and dofetilide. Symbols indicate mean values ± sem from at least 2 independent plates. The data is plotted as a function of the experimental concentration divided by the total Cmax^8^. Asterisks indicate that the change in relative surface expression is significantly different from the relative surface expression of the lower concentration tested for each drug.

We tested concentrations of bepridil and vanoxerine at 10 to 30 times the total plasma concentrations. These concentrations were at least one order of magnitude smaller than the concentrations reached in rabbit myocardium after direct perfusion with vanoxerine or bepridil which were concentrated 430^20^ and 914^21^ times above the plasma concentrations. Figure 5 shows the percentage changes in surface hERG expression relative to vehicle controls as a function of the experimental concentration normalized to the total effective therapeutic plasma concentration. In the presence of bepridil hERG surface expression was decreased significantly to 38% at ~10 times the total plasma concentration. Over the same concentration range, vanoxerine increased surface expression of wild type hERG by 30% and dofetilide by 26%. The increase in hERG surface expression by dofetilide is consistent with the increased trafficking efficiency observed by Varkevisser *et al.* (2013)^22^. Verapamil did not affect hERG surface expression.

## Discussion

We showed that vanoxerine, a potent hERG blocker, may be a safe, effective antiarrhythmic drug because it produces Multiple Ion Channel Effects (MICE) that offset the hERG block. Action potentials measured experimentally in iPSC-derived cardiomyocytes reflected the drug-induced changes in MICE currents that were observed in cell lines heterologously expressing hERG, hCav 1.2 and hNav 1.5. These currents are also expressed in iPSC cardiomyocytes^23^,^24^,^25^. The simulated human left ventricular action potentials compared favorably with the experimental action potentials.

For dofetilide the selective block of hERG accounts for the proarrhythmia markers we observed experimentally and in simulations and by extension the drug’s torsadogenicity. For verapamil and vanoxerine MICE effects offset hERG block, account for the experimental and simulated action potentials and explain their non-torsadogenicity. For the torsadogenic MICE drug bepridil however the anticipated proarrhythmia markers were not observed during acute exposure either experimentally or in simulations. We considered longer term exposure as a possibility since we showed previously that bepridil inhibited hERG trafficking whereas verapamil and vanoxerine did not and we have confirmed these results in this study. We were unable to measure the electrophysiological effects of prolonged exposure in iPSC cardiomyocytes due to excessive depolarization and cytotoxicity perhaps due to excessive intracellular accumulation. Additional torsadogenicity of bepridil may also be related to an increase in the surface expression of hNav 1.5 channels^26^, anti-calmodulin activity^27^ and promotion of phospholipidosis^28^.

Vanoxerine block of hCav 1.2 and hNav 1.5 was more potent and hERG less potent than reported previously^4^. We ascribe the differences to our quantitative profiling method, namely the use of a more physiological step-ramp voltage protocol, similarities of cell preparations (e.g., hCav 1.2 channels expressed heterologously in cell lines as for hERG and Nav1.5 rather than guinea pig cardiomyocytes) and use of the perforated patch clamp configuration instead of whole cell patch clamp for calcium currents.

Previously we showed a limited effect of vanoxerine on the APD recorded in the arterially-perfused canine wedge preparation^4^ compared to the results recorded presently. This discordance can be explained by lack of exposure to the total concentration of drug in the wedge preparation. We hypothesized that the difference was due to non-specific tissue binding and our hypothesis was tested by *in silico* modeling based on the Decker-Rudy model of a canine epicardial cell^29^ as shown in Supplementary Figure 6.

Quantitative profiling of verapamil resulted in less potent hERG block compared to previous reports which used step pulses much longer (>1 s) than would occur physiologically^30^,^31^,^32^,^33^. The result was consistent with our SC-CMAP studies where prolongation of APD90 only appeared at 1 μM free concentration. This observation was validated in our simulations as shown in Supplementary Figure 5. It has been reported that moxifloxacin, dofetilide and cisapride show less potent hERG block when tested using ventricular-like action potential waveforms^25^,^26^.

We consider our proarrhythmia analysis in relation to clinical results by reference to Fig. 2 in which blocking potencies are compared with free plasma concentrations. Dofetilide produced EADs in SC-CMAPs at 10 nM *in vitro* whereas vanoxerine at 100 nM produced none. Notably, the arrhythmogenic *in vitro* concentration of dofetilide at 10 nM is 5 times the *in vivo* exposure, whereas vanoxerine did not induce arrhythmias up to 100 nM at least 12 times the estimated free *in vivo* exposure. It is possible that the increase in hERG surface expression by vanoxerine could mitigate its channel blocking properties.

A limitation of this study is that experiments using cell lines in which ion channels were expressed heterologously were done at room rather than physiological temperature because run-down of the calcium current was excessive at PT. Nevertheless the IC50s measured at room temperature account for the simulated results at 37 °C and the experimental results at 35 °C using hSC-CMAPs. An additional limitation is that we could not accommodate KvLQT1/minK current measurements at 1 Hz. However, previous experiments at 15 sec intervals had an IC50 of 2,900 nM far from the IC50s reported presently for block of hERG, Cav 1.2 and Nav 1.5 which were 9.3, 16.2 and 34.6 nM respectively. Finally, we used ATX II-activated hNav1.5 currents at 30 nM as a surrogate of the late sodium current^34^. This concentration is larger than the 20 nM EC50 measured in rabbit cardiomyocytes^35^. ATX II-activated currents show pharmacological properties comparable to late sodium currents in a number of species. We observed that ranolazine blocks ATX II- activated currents with an IC50 of 5,312 ± 633 nM (95% CI: 3,904–6,787) and a Hill coefficient of 0.96 ± 0.12 (95% CI: 0.73–1.24). These values compare with the 5,900 nM IC50 measured in isolated canine ventricular myocytes^36^ and the 7,280 nM IC50 measured at 1 Hz in a mutant human sodium channel^37^. Also, ATX II-activated late hNav1.5 currents are blocked by amiodarone with a 3,000 nM IC50^38^, a value comparable to the 6,700 nM IC50^39^ measured in late sodium currents in human cardiomyocytes. Note that vanoxerine block of ATX II-activated hNav1.5 currents is at least 56 times more potent than ranolazine.

Taken together the MICE of vanoxerine in a background of normal or increased hERG surface expression may account for the absence of proarrhythmic effects and termination of AF/AFL in clinical trials^3^ as predicted by our nonclinical hypothesis. Our speculation concerning the safety of vanoxerine simply put is as follows. Block of calcium and sodium currents evidenced by the reduction in amplitude and plateau of the action potential prevents EADs due to reactivation of inactivated calcium and sodium channels which are thought to be causative^40^,^41^. Despite prolongation of the AP associated with termination of AF/AFL the absence of EADs accounts for the lack of serious ventricular arrhythmias and the drug’s safety. On the efficacy side without regard to hypotheses of multiple wavelets or driving rotors^42^ the very same MICE of vanoxerine prolong refractoriness due to hERG block, abolish propagation due to sodium and calcium currents and terminate the reentry pathways of AF and AFL.

Quantitative profiling as it relates to CiPA relies on the concept that cardiac risk assessment can be ascertained from acute and direct effects on channel gating and/or pore block. Drugs like bepridil that inhibit trafficking of the channel protein add a new layer of complexity to nonclinical risk assessment that should be investigated. Thus quantitative profiling and trafficking effects should be considered as complementary in nonclinical cardiac risk assessment.

## Methods

### Cells

The Kv11.1 channel (hERG), Nav 1.5 channel (hNav 1.5 channel) and hKir 2.1 channel were stably expressed in HEK 293 cells. The Cav 1.2 channel (hCav1.2/b2/a2d channel) was stably expressed in CHO cells. All cell lines were from ChanTest.

### Voltage clamp experiments

We measured hERG, peak and late hNav 1.5 and hKir 2.1 currents using the fast whole cell configuration of the patch clamp technique. To investigate hCav 1.2 currents we used the perforated patch clamp configuration with amphotericin B as the perforating agent^43^. Ion channel currents were measured using an Axopatch 200B or a MultiClamp 700B amplifier. All experiments were performed at ambient temperature. The extracellular solution for the three channels contained (in mM): 137 NaCl, 4 KCl, 1.8 CaCl_2_, 1 MgCl_2_, 1; 10 HEPES, 10 Glucose, pH adjusted to 7.4 with NaOH. The intracellular solution to study hERG currents contained: 130 K-aspartate, 5 MgCl_2_, 5 EGTA, 4 Tris_2_-ATP, 10 HEPES, pH adjusted to 7.2 with KOH. The intracellular solution used in the perforated patch clamp configuration contained: 130 CsCl, 5 MgCl_2_, 5 EGTA, 10 HEPES, pH adjusted to 7.2 with CsOH. The intracellular solution to study Nav 1.5 currents contained: 130 Cs-aspartate, 5 MgCl_2_, 5 EGTA, 10 HEPES, Na2-ATP, Tris-GTP pH adjusted to 7.2 with CsOH. We used 30 nM ATX II, a toxin derived from *Anemonia sulcata* to activate the late hNav1.5 current. Leak currents were recorded in the presence of specific blockers including 1 μM E-4031 to block hERG currents, 25 nM nifedipine and 30 μM Cd^2+^ to block hCav 1.2 currents, 2 mM lidocaine to block peak and late hNav 1.5 currents and 2 mM Ba^2+^ to block Kir 2.1 currents.

The voltage protocol used to study hERG currents consisted of a 220 ms step to +30 mV followed by a 200 ms ramp to −80 mV. Peak hERG currents were measured during the ramp. For hCav 1.2 currents the voltage protocol consisted of a 40 ms step to 0 mV, a 180 ms step to +30 mV and a 200 ms ramp to −80 mV. Peak hCav 1.2 currents were measured at 0 mV. For Nav 1.5 currents including peak (peak hNav 1.5) and late sodium current (late hNav 1.5) the voltage protocol consisted of a 40 ms step to −15 mV, a 180 ms step to +30 mV and a 200 ms ramp to −80 mV. Peak hNav1.5 currents were measured at −15 mV whereas peak late hNav1.5 currents except for verapamil were measured during the ramp. The holding potential in all protocols was −80 mV. Step-ramp protocols were delivered at 1 Hz. In the case of verapamil we used a voltage protocol consisting of a 200 ms pulse to −120 mV, a 5 ms pulse to +50 mV followed by a 400 ms pulse to −30 mV.

For data analysis currents were leak-subtracted and corrected for rundown assuming a linear current decay determined by the slope measured at baseline and after reaching steady state during drug exposure.

### Current clamp experiments

Experiments were performed at 35 °C using the gramicidin perforated patch clamp configuration^44^. The extracellular solution contained (in mM): 150 NaCl, KCl, 1.8 CaCl_2_, 1 MgCl_2_, 15 HEPES, 15 Glucose, 1 Na-pyruvate pH adjusted to 7.4 with NaOH. The intracellular solution for the perforated patch contained: 150 KCl, 5 NaCl, 2 CaCl_2_, 5 Mg-ATP, 5 EGTA, 10 HEPES pH adjusted to 7.2 with KOH. Action potentials were recorded from human iPSC-derived cardiomyocytes (Axiogenesis Cor.4U®) plated on Geltrex®-coated 35-mm plastic dishes in clusters of approximately 20,000 cells. Before plating to the 35 mm dishes used for testing, frozen vials of cells were thawed per manufacturer instructions, pre-plated onto 0.2% gelatin or Geltrex®-coated 6 well plates (approximately 800,000 cells/well), and cultured for approximately 2 to 21 days. Cells were beating spontaneously between 0.5 Hz and 1.5 Hz. Only cells with maximal upstroke velocities >100 V/s were tested.

Data acquisition was performed using a MultiClamp 700B amplifier, a Digidata 1320A digitizer, and pCLAMP 10.2 software (MDS Inc., Union City, CA). Analog signals were low-pass filtered at 10 kHz before digitization at 50 kHz (DT3010 Data translation, Marlboro, MA), and stored on hard disk using a PC-compatible computer controlled by Notocord-Hem 4.3 software (Notocord Systems SA, Croissy, France). Data analysis was performed using Notocord-Hem 4.3 software and Microsoft Excel.

The average responses of at least five recorded action potentials at each concentration were analyzed. The following parameters were quantitated: APD30, APD60 and APD90 (action potential duration at 30%, 60% and 90% repolarization, respectively, ms), MDP (maximum diastolic membrane potential, mV), APA (action potential amplitude, mV), overshoot (mV) and upstroke velocity (Vmax; V/s) and period. Changes in APD30, APD60, APD90, APA, Vmax and period are presented as percent change (Δ%) from baseline at each concentration. MDP and overshoot data are presented as change in membrane potential (ΔmV) from baseline. Data are reported as mean ± s.e.m. Pooled data were tabulated for the baseline control and each test article concentration.

### Simulations

We used the O’Hara-Rudy model of the endocardial human cardiomyocyte^12^ to implement MICE parameters of drug effects on the cardiac action potential. Drugs effects were simulated using the conductance-block method^45^ in which original model conductances (g_control_) of the currents under investigation are decreased by factors that depend on block potencies obtained under steady state conditions and parameterized by the IC50s and Hill coefficients (h) according to equation (1).


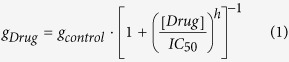


Simulations were performed in two steps. First the system was allowed to reach steady state after 1000 iterations at 1 Hz. Then, conductances were adjusted according to the free drug concentrations and a second 1000 iteration run was allowed to reach a new steady state. Action potentials were elicited using 0.5 ms, −80 μA/μF pulses.

### Chemiluminescence Detection of hERG Protein at the Cell Surface

The effect of the drugs on the surface expression of hERG was investigated using the hERG-lite technique based on the detection of an hemagglutinin (HA) tag inserted into the extracellular loop of the hERG channel between transmembrane domains S1 and S2 46. This tag had no effect on channel function. Stably transfected HEK/hERG WT HA cells were plated at 40,000 cells/well in a 96-well plate. After overnight incubation with vanoxerine, bepridil, dofetilide and verapamil, cells were fixed with ice-cold 4% paraformaldehyde, blocked by incubation with 1% goat serum, and incubated for 1 h with rat anti-HA antibody (Roche Diagnostics). After washing, horseradish peroxidase-conjugated goat anti-rat IgG (Jackson ImmunoResearch Laboratories Inc., West Grove, PA) and a dsDNA stain (SYBR Green, Molecular Probes, Eugene, OR; or Hoechst33342, Thermo Fisher Scientific, Waltham, MA) were added for 1 h (Myers, 1998; Margeta-Mitrovic *et al.*, 2000). Fluorescence was measured to determine cell numbers. Chemiluminescent signals were developed using SuperSignal (Pierce Chemical) and captured in a luminometer. Fluorescence and luminescence values from drug-treated wells were normalized to values from a standard curve of wells seeded with 10,000–50,000 cells and incubated overnight with vehicle (n ≥ 12 wells per plate). This allowed the relative surface expression of hERG from drug-treated wells to be determined for wells where up to 75% of the cells were lost during the assay (relative to vehicle-treated wells), for example due to cytotoxic or cytostatic effects.

### Statistical Analysis

For voltage-clamp experiments, we used JMP software (Version 9, SAS Institute, Cary, NC) to fit the data to single binding isotherms parameterized by IC50s and Hill coefficients. The fitted parameters are reported as mean ± s.e.m. and 95% upper and lower confidence intervals. We used the F-test to assess the equality of variances and the t-test to assess baseline differences between vanoxerine and dofetilide. We used a two-sided paired t-test to assess the effect of each drug. Changes in action potential parameters were evaluated using a two-sided paired t-test (Microsoft Excel) to determine whether the change from baseline observed after equilibration in each test article concentration was statistically significant (*P* < 0.05) from that observed in baseline control.

## Additional Information

**How to cite this article**: Obejero-Paz, C. A. *et al.* Quantitative Profiling of the Effects of Vanoxerine on Human Cardiac Ion Channels and its Application to Cardiac Risk. *Sci. Rep.* **5**, 17623; doi: 10.1038/srep17623 (2015).

## Supplementary Material

Supplementary Information

## Figures and Tables

**Table 1:** IC50 and Hill coefficients used in simulations.

Drug	hCav1.2	hERG	hNav1.5 peak	hNav1.5 late	IKs	Ito	Kir2.1
Vanoxerine IC50 (nM)	16.2 (11.0–22.0)	9.3 (7.2–11.8)	34.6 (23.4–48.8)	85.2 (72.9–99.9)	2,900	2,000	98,124
h	0.63 (0.44–0.85)	1.11 (0.84–1.48)	0.97 (0.57–1.48)	1.62 (1.30–2.09)	1.00	1.00	1.00
Bepridil IC50 (nM)	175 (139–221)	73.8 (68–80)	517 (438–604)	411 (353–477)	6,156 (4,162–8,897)	na	66,536
h	1.08 (0.83–1.42)	1.33 (1.19–1.49)	1.14 (0.91–1.41)	1.72 (1.29–2.26)	2.23 (1.13 - na)	na	1
Dofetilide IC50 (nM)	26,700	5.2 (3.8–6.8)	147,900	na	415,750	na	98,833
h	1.00	0.97 (0.69–1.37)	1.00	na	1.00	na	1.00
Verapamil IC50 (nM)	79.4 (51.6–113.5)	831 (717–926)	7,221 (5,527–9,184)	6,094	65,587	na	9,033
h	0.69 (0.48–0.94)	1.17 (1.01–1.36)	0.95 (0.74–1.21)	1.24	0.92	na	1.00

hERG, hCav 1.2, peak and late hNav 1.5 and hKir 2.1 data used in vanoxerine simulations are from this study. IKs and Ito data are from Lacerda *et al.* (2010)^4^. hERG data used in dofetilide simulations are from this study whereas hCav 1.2 and peak hNav 1.5 data are from Kramer *et al.* (2013)^8^. hERG, hCav 1.2, peak and late Nav 1.5 data used in the verapamil simulations are from this study. IKs and hKir 2.1 data for dofetilide, bepridil and verapamil are unpublished results. Number in parentheses indicates lower and upper 95% confidence intervals. na: not available.

## References

[b1] Søgaard U. *et al.* A tolerance study of single and multiple dosing of the selective dopamine uptake inhibitor GBR 12909 in healthy subjects. Int. Clin. Psychopharmacol. 5, 237–251 (1990). 2150527 10.1097/00004850-199010000-00001

[b2] Preti A. Vanoxerine National Institute on Drug Abuse. Curr. Opin. Investig. Drugs. 1, 241–251 (2000). 11249581

[b3] Dittrich H. C. *et al.* COR-ART: A multicenter, randomized, double-blind, placebo- controlled dose ranging study to evaluate single oral doses of vanoxerine for conversion of recent onset atrial fibrillation or flutter to normal sinus rhythm. Heart Rhythm. 12, 1105–1112 (2015). 25684233 10.1016/j.hrthm.2015.02.014

[b4] Lacerda A. E., Kuryshev Y. A., Yan G. X., Waldo A. L. & Brown A. M. Vanoxerine: cellular mechanism of a new antiarrhythmic. J. Cardiovasc. Electrophysiol. 21, 301–310 (2010). 19817928 10.1111/j.1540-8167.2009.01623.x PMC3107714

[b5] ICH. Harmonised Tripartite Guideline S7B. Non-clinical evaluation of the potential for delayed ventricular repolarization (QT interval prolongation) by human pharmaceuticals. Step 4 Version, May 2005. Available at: http://www.ich.org/fileadmin/Public_Web_Site/ICH_Products/Guidelines/Safety/S7B/Step4/S7B_ Guideline.pdf. (Accessed 20 February 2015).

[b6] ICH. Harmonised Tripartite Guideline E14. Clinical evaluation of QT/QTc interval prolongation and proarrhythmic potential for nonantiarrhythmic drugs. Step 4 Version, May 2005. Available at: http://www.ich.org/fileadmin/Public_Web_Site/ICH_Products/Guidelines/Efficacy/E14/E14_Guid eline.pdf. (Accessed 20 February 2015).

[b7] Redfern W. S. *et al.* Relationships between preclinical cardiac electrophysiology, clinical QT interval prolongation and torsade de pointes for a broad range of drugs: evidence for a provisional safety margin in drug development. Cardiovasc. Res. 58, 32–45 (2003). 12667944 10.1016/s0008-6363(02)00846-5

[b8] Kramer J. *et al.* MICE models: superior to the hERG model in predicting torsade de pointes. Sci. Rep. 3, 2100 (2013). 23812503 10.1038/srep02100 PMC3696896

[b9] Matsumoto N. *et al.* Vanoxerine, a new drug for terminating atrial fibrillation and flutter. J. Cardiovasc. Electrophysiol. 21, 311–319 (2010). 19817929 10.1111/j.1540-8167.2009.01622.x

[b10] Cakulev I. *et al.* Oral vanoxerine prevents reinduction of atrial tachyarrhythmias: preliminary results. J. Cardiovasc. Electrophysiol. 22, 1266–1273 (2011). 21615815 10.1111/j.1540-8167.2011.02098.x PMC3172341

[b11] Rampe D. & Brown A. M. A history of the role of the hERG channel in cardiac risk assessment. J. Pharmacol. Toxicol. Methods. 68, 13–22 (2013). 23538024 10.1016/j.vascn.2013.03.005

[b12] O’Hara T., Virág L., Varró A. & Rudy Y. Simulation of the undiseased human cardiac ventricular action potential: model formulation and experimental validation. PLoS. Comput. Biol. 7, e1002061 (2011). 21637795 10.1371/journal.pcbi.1002061 PMC3102752

[b13] Suttorp M. J., Kingma J. H., Lie-A-Huen L. & Mast E. G. Intravenous flecainide versus verapamil for acute conversion of paroxysmal atrial fibrillation or flutter to sinus rhythm. Am. J. Cardiol. 63, 693–696 (1989). 2493733 10.1016/0002-9149(89)90253-1

[b14] Sager P. T., Gintant G., Turner J. R., Pettit S. & Stockbridge N. Rechanneling the cardiac proarrhythmia safety paradigm: a meeting report from the Cardiac Safety Research Consortium. Am. Heart J. 167, 292–300 (2014). 24576511 10.1016/j.ahj.2013.11.004

[b15] De Bruin M. L., Pettersson M., Meyboom R. H., Hoes A. W. & Leufkens H. G. Anti- hERG activity and the risk of drug-induced arrhythmias and sudden death. Eur. Heart J. 26, 590–597 (2005). 15637086 10.1093/eurheartj/ehi092

[b16] Mirams G. R. *et al.* Simulation of multiple ion channel block provides improved early prediction of compounds’ clinical torsadogenic risk. Cardiovasc. Res. 91, 53–61 (2011). 21300721 10.1093/cvr/cvr044 PMC3112019

[b17] Mirams G. R. *et al.* Prediction of thorough QT study results using action potential simulations based on ion channel screens. J. Pharmacol. Toxicol. Methods. 70, 246–254 (2014). 25087753 10.1016/j.vascn.2014.07.002 PMC4266452

[b18] Coumel P. Safety of bepridil: from review of the European data. Am. J. Cardiol. 69, 75D–78D (1992). 10.1016/0002-9149(92)90963-y 1553894

[b19] Wible B. A. *et al.* HERG-Lite: a novel comprehensive high-throughput screen for drug-induced hERG risk. J. Pharmacol. Toxicol. Methods. 52, 136–145 (2005). 15950494 10.1016/j.vascn.2005.03.008

[b20] Nielsen-Kudsk F., Siggaard C., Nielsen C. B. & Mellemkjaer S. Myocardial accumulation kinetics and pharmacodynamics in the isolated rabbit heart of a new inhibitor of dopamine reuptake, GBR 12909. Pharmacol. Toxicol. 66, 197–202 (1990). 2139724 10.1111/j.1600-0773.1990.tb00732.x

[b21] Nielsen-Kudsk F., Askholt J. & Jakobsen P. Bepridil, myocardial accumulation kinetics and dynamic effects in the isolated rabbit heart. Pharmacol. Toxicol. 63, 122–128 (1988). 3263633 10.1111/j.1600-0773.1988.tb00923.x

[b22] Varkevisser R. *et al.* Structure-activity relationships of pentamidine-affected ion channel trafficking and dofetilide mediated rescue. Br. J. Pharmacol. 169, 1322–1334 (2013). 23586323 10.1111/bph.12208 PMC3831711

[b23] Ma J. *et al.* High purity human-induced pluripotent stem cell-derived cardiomyocytes: electrophysiological properties of action potentials and ionic currents. Am. J. Physiol. Heart Circ. Physiol. 301, H2006–H2017 (2011). 21890694 10.1152/ajpheart.00694.2011 PMC4116414

[b24] Scheel O. *et al.* Action potential characterization of human induced pluripotent stem cell-derived cardiomyocytes using automated patch-clamp technology. Assay Drug. Dev. Technol. 12, 457–469 (2014). 25353059 10.1089/adt.2014.601

[b25] Kirsch G., Obejero-Paz C. & Bruening-Wright A. Functional characterization of human stem cell-derived cardiomyocytes. Curr. Protoc. Pharmacol. 64, 11–12 (2014). 10.1002/0471141755.ph1112s64 PMC4138727 25152802

[b26] Kang L. *et al.* Bepridil up-regulates cardiac Na^+^ channels as a long-term effect by blunting proteasome signals through inhibition of calmodulin activity. Br. J. Pharmacol. 157, 404–414 (2009). 19371335 10.1111/j.1476-5381.2009.00174.x PMC2707987

[b27] Itoh H., Ishikawa T. & Hidaka H. Effects on calmodulin of bepridil, an antianginal agent. J. Pharmacol. Exp. Ther. 230, 737–741 (1984). 6088765

[b28] Muehlbacher M., Tripal P., Roas F. & Kornhuber J. Identification of drugs inducing phospholipidosis by novel *in vitro* data. ChemMedChem. 7, 1925–1934 (2012). 22945602 10.1002/cmdc.201200306 PMC3533795

[b29] Decker K. F. & Rudy Y. Ionic mechanisms of electrophysiological heterogeneity and conduction block in the infarct border zone. Am. J. Physiol. Heart Circ. Physiol. 299, 1588–1597 (2010). 10.1152/ajpheart.00362.2010 PMC2993197 20709867

[b30] Kirsch G. E. *et al.* Variability in the measurement of hERG potassium channel inhibition: effects of temperature and stimulus pattern. J. Pharmacol. Toxicol. Methods. 50, 93–101 (2004). 15385083 10.1016/j.vascn.2004.06.003

[b31] Zhang S., Zhou Z., Gong Q., Makielski J. C. & January C. T. Mechanism of block and identification of the verapamil binding domain to HERG potassium channels. Circ. Res. 84, 989–998 (1999). 10325236 10.1161/01.res.84.9.989

[b32] Alexandrou A. J. *et al.* Mechanism of hERG K+ channel blockade by the fluoroquinolone antibiotic moxifloxacin. Br. J. Pharmacol. 147, 905–916 (2006). 16474415 10.1038/sj.bjp.0706678 PMC1760709

[b33] Milnes J. T., Witchel H. J., Leaney J. L., Leishman D. J. & Hancox J. C. Investigating dynamic protocol-dependence of hERG potassium channel inhibition at 37 °C: Cisapride versus dofetilide. J. Pharmacol. Toxicol. Methods. 61, 178–191 (2010). 20172036 10.1016/j.vascn.2010.02.007

[b34] Song Y., Shryock J. C., Wu L. & Belardinelli L. Antagonism by ranolazine of the pro- arrhythmic effects of increasing late INa in guinea pig ventricular myocytes. J. Cardiovasc. Pharmacol. 44, 192–199 (2004). 15243300 10.1097/00005344-200408000-00008

[b35] Luo A. *et al.* Larger late sodium current density as well as greater sensitivities to ATX II and ranolazine in rabbit left atrial than left ventricular myocytes. Am. J. Physiol. Heart Circ. Physiol. 306, H455–H461 (2014). 24322614 10.1152/ajpheart.00727.2013

[b36] Antzelevitch C. *et al.* Electrophysiological effects of ranolazine, a novel antianginal agent with antiarrhythmic properties. Circulation. 110, 904–910 (2004). 15302796 10.1161/01.CIR.0000139333.83620.5D PMC1513623

[b37] Rajamani S., El-Bizri N., Shryock J. C., Makielski J. C. & Belardinelli L. Use- dependent block of cardiac late Na^+^ current by ranolazine. Heart Rhythm. 6, 1625–1631 (2009). 19879541 10.1016/j.hrthm.2009.07.042 PMC2879577

[b38] Wu L. *et al.* Augmentation of late sodium current unmasks the proarrhythmic effects of amiodarone. Cardiovasc. Res. 77, 481–488 (2008). 18006430 10.1093/cvr/cvm069 PMC2365898

[b39] Maltsev V. A., Sabbah H. N. & Undrovinas A. I. Late sodium current is a novel target for amiodarone: studies in failing human myocardium. J. Mol. Cell. Cardiol. 33, 923–932 (2001). 11343415 10.1006/jmcc.2001.1355

[b40] January C. T. & Riddle J. M. Early afterdepolarizations: mechanism of induction and block. A role for L-type Ca2+ current. Circ. Res. 64, 977–990 (1989). 2468430 10.1161/01.res.64.5.977

[b41] Qu Z. *et al.* Early afterdepolarizations in cardiac myocytes: beyond reduced repolarization reserve. Cardiovasc. Res. 99, 6–15 (2013). 23619423 10.1093/cvr/cvt104 PMC3687754

[b42] Pandit S. V. & Jalife J. Rotors and the dynamics of cardiac fibrillation. Circ. Res. 112, 849–862 (2013). 23449547 10.1161/CIRCRESAHA.111.300158 PMC3650644

[b43] Rae J., Cooper K., Gates P. & Watsky M. Low access resistance perforated patch recordings using amphotericin B. J. Neurosci. Methods. 37, 15–26 (1991). 2072734 10.1016/0165-0270(91)90017-t

[b44] Tajima Y., Ono K. & Akaike N. Perforated patch-clamp recording in cardiac myocytes using cation-selective ionophore gramicidin. Am. J. Physiol. 271, C524–C532 (1996). 8769991 10.1152/ajpcell.1996.271.2.C524

[b45] Brennan T., Fink M. & Rodriguez B. Multiscale modelling of drug-induced effects on cardiac electrophysiological activity. Eur. J. Pharm. Sci. 36, 62–77 (2009). 19061955 10.1016/j.ejps.2008.09.013

[b46] Ficker E., Dennis A. T., Wang L. & Brown A. M. Role of the cytosolic chaperones Hsp70 and Hsp90 in maturation of the cardiac potassium channel HERG. Circ Res. 92, e87–e100 (2003). 12775586 10.1161/01.RES.0000079028.31393.15

