# Supplementary material for: Quantitative Profiling of the Effects of Vanoxerine on Human Cardiac Ion Channels and its Application to Cardiac Risk
Source: Sci Rep. 2015 Nov 30;5:17623. doi: 10.1038/srep17623 (PMC4663487; doi:10.1038/srep17623)
Supplement: Supplementary Information [file srep17623-s1.pdf]

Supplementary Materials

Quantitative Profiling of the Effects of Vanoxerine on Human Cardiac Ion Channels and its Application to Cardiac Risk

Carlos A. Obejero-Paz[1], Andrew Bruening-Wright[1], James Kramer[1],

Peter Hawryluk[1], Milos Tatalovic[1], Howard C. Dittrich[2], and Arthur M. Brown[1,2*]

Supplementary Table S1. Effect of vanoxerine on action potential parameters

Baseline parameters										
Parameter	APD30 ms	APD60 ms	APD90 ms	MDP mV	APA mV	Over Shoot mV	Vmax V/s	Period ms	EADs	N
Mean ± s.e.m	193 ± 4	245 ± 7	283 ± 9	-83 ± 1	117 ± 0.4	34 ± 0.4	186 ± 33	1,232 ± 179	0/3	3
**Change from baseline**										
Concentration (nM)	Δ%	Δ%	Δ%	ΔmV	Δ%	ΔmV	Δ%	Δ%	EADs	N
10	2.9 ± 1.1	6.7 ± 0.8*	8.8 ± 0.5*	0.2 ± 0.5	-0.3 ± 0.6	-0.1 ± 0.4	3.4 ± 3.0	23.4 ± 16.9	0/3	3
30	9.2 ± 0.9*	23.5 ± 2.1*	32.5 ± 3.5*	2.2 ± 0.5*	-2.6 ± 1.6	-0.7 ± 2.0	-2.1 ± 4.7	55.5 ± 19.8	0/3	3
100	13.2 ± 2.9*	56.9 ± 1.9*	110.6 ± 1.7*	3.9 ± 1.2	-5.2 ± 1.0*	-2.2 ± 1.8	-25.6 ± 5.5	94.1 ± 25.8*	0/3	3

APDs 30, 60 and 90 indicate duration of the action potential from the point of maximal dV/dt in the upstroke to 30%, 60% and 90% repolarization; MDP: maximal diastolic potential; APA: action potential amplitude; Overshoot: maximal depolarization; Vmax: maximal depolarization rate (dV/dt) during the upstroke of the action potential; Period: time between two action potentials. EADs: early after depolarization. The ratio indicates the fraction of cells showing EADs. N: number of cells. Asterisks indicate significant difference with respect to baseline at $p < 0.05$.

Supplementary Table S2. Effect of bepridil on action potential parameters

Baseline parameters										
Parameter	APD30 ms	APD60 ms	APD90 ms	MDP mV	APA mV	Over Shoot mV	Vmax V/s	Period ms	EADs	N
Mean ± s.e.m.	165 ± 27	213 ± 36	246 ± 40	-82 ± 1	114 ± 2	33 ± 2	185 ± 21	985 ± 108	0/5	5
**Change from baseline**										
Concentration (nM)	Δ%	Δ%	Δ%	ΔmV	Δ%	ΔmV	Δ%	Δ%	EADs	N
30	2.7 ± 0.3*	5.5 ± 0.8*	6.8 ± 0.9*	0.6 ± 0.4	-0.6 ± 0.4	0 ± 1.2	-7.3 ± 4.7	3.3 ± 0.8	0/5	5
100	11.4 ± 1.1*	24.6 ± 2.5*	31.1 ± 2.8*	1.4 ± 0.3*	-1.7 ± 0.6	-1.7 ± 1.2	-10.6 ± 5.8	7.8 ± 6.0	0/5	5
300	19.8 ± 5.8*	52.5 ± 1.6*	90.9 ± 3.7*	6.8 ± 0.9*	-8.5 ± 3.3*	-8.7 ± 9.0	-54.8 ± 15.2*	34.2 ± 4.8*	0/4	4

APDs 30, 60 and 90 indicates duration of the action potential from the point of maximal dV/dt in the upstroke to 30%, 60% and 90% repolarization; MDP: maximal diastolic potential; APA: action potential amplitude; Overshoot: maximal depolarization; Vmax: maximal depolarization rate (dV/dt) during the upstroke of the action potential; Period: time between two action potentials. EADs: early after depolarization. The ratio indicates the fraction of cells showing EADs. N: number of cells. NM: not measured due to the presence of arrhythmias. Asterisks indicate significant difference with respect to baseline at $p < 0.05$.

Supplementary Table S3. Effect of verapamil on action potential parameters

Baseline parameters										
Parameter	APD30 ms	APD60 ms	APD90 ms	MDP mV	APA mV	Over Shoot mV	Vmax V/s	Period ms	EADs	N
Mean ± s.e.m.	190 ± 8	247 ± 8	286 ± 9	-83 ± 1	118 ± 1	35 ± 1	183 ± 38	1,071 ± 240	0/3	3
**Change from baseline**										
Concentration (nM)	Δ%	Δ%	Δ%	ΔmV	Δ%	ΔmV	Δ%	Δ%	EADs	N
30	-5.8 ± 1.0*	-5.3 ± 0.6*	-4.3 ± 0.5*	-0.1 ± 0.5	-3.3 ± 1.1	-4.0 ± 1.1	-6.9 ± 2.4	2.4 ± 2.4	0/3	3
100	-31.4 ± 2.4*	-27.0 ± 1.1*	-22.3 ± 1.1*	0.9 ± 1.0	-7.7 ± 3.1	-8.3 ± 2.7	-8.3 ± 3.1	-16.4 ± 1.5*	0/3	3
300	-53.2 ± 5.6*	-43.3 ± 2.5*	-33.5 ±1.9*	1.1 ± 1.1	-11.8 ± 4.4	-12.8 ± 4.7	-9.3 ± 3.3*	-28.0 ± 1.1*	0/3	3
1000	-69.1 ± 6.7*	-50.1 ± 2.9*	-27.4 ±1.7*	2.8 ± 1.0	-18.1 ± 6.3	-18.5 ± 6.6	-18.4 ± 4.2*	-23.5 ± 3.3*	0/3	3

APDs 30, 60 and 90 indicates duration of the action potential from the point of maximal dV/dt in the upstroke to 30%, 60% and 90% repolarization; MDP: maximal diastolic potential; APA: action potential amplitude; Overshoot: maximal depolarization; Vmax: maximal depolarization rate (dV/dt) during the upstroke of the action potential; Period: time between two action potentials. EADs: early after depolarization. The ratio indicates the fraction of cells showing EADs. N: number of cells. NM: not measured due to the presence of arrhythmias. Asterisks indicate significant difference with respect to baseline at $p < 0.05$.

Supplementary Table S4. Effect of dofetilide on action potential parameters

Baseline parameters										
Parameter	APD30 ms	APD60 ms	APD90 ms	MDP mV	APA mV	Over Shoot mV	Vmax V/s	Period ms	EADs	N
Mean ± s.e.m.	232 ± 16	298 ± 27	338 ± 32	-83 ± 1	119 ± 1	37 ± 1	119 ± 8	1,503 ± 240	0/3	3
**Change from baseline**										
Concentration (nM)	Δ%	Δ%	Δ%	ΔmV	Δ%	ΔmV	Δ%	Δ%	EADs	N
3	5.8 ± 1.9	19.8 ± 3.9	24.0 ± 4.1	-0.01 ± 1.4	-0.7 ± 0.1*	-2.4 ± 3.3	1.8 ± 2.1	-4.6 ± 4.5	0/3	3
10	20.3 ± 6.8	77.4 ± 5.5*	90.4 ± 7.4*	-0.2 ± 1.1	-1.2 ± 0.4	-3.7 ± 3.0	-18.2 ± 3.8	16.2 ± 7.8	2/3	3
30	NM	NM	NM	NM	NM	NM	NM	NM	3/3	3
100	NM	NM	NM	NM	NM	NM	NM	NM	3/3	3

APDs 30, 60 and 90 indicates duration of the action potential from the point of maximal dV/dt in the upstroke to 30%, 60% and 90% repolarization; MDP: maximal diastolic potential; APA: action potential amplitude; Overshoot: maximal depolarization; Vmax: maximal depolarization rate (dV/dt) during the upstroke of the action potential; Period: time between two action potentials. EADs: early after depolarization. The ratio indicates the fraction of cells showing EADs. N: number of cells. NM: not measured due to the presence of arrhythmias. Asterisks indicate significant difference with respect to baseline at $p < 0.05$.

Supplementary Figure 1. Time course of the experiments shown in text Figure 1

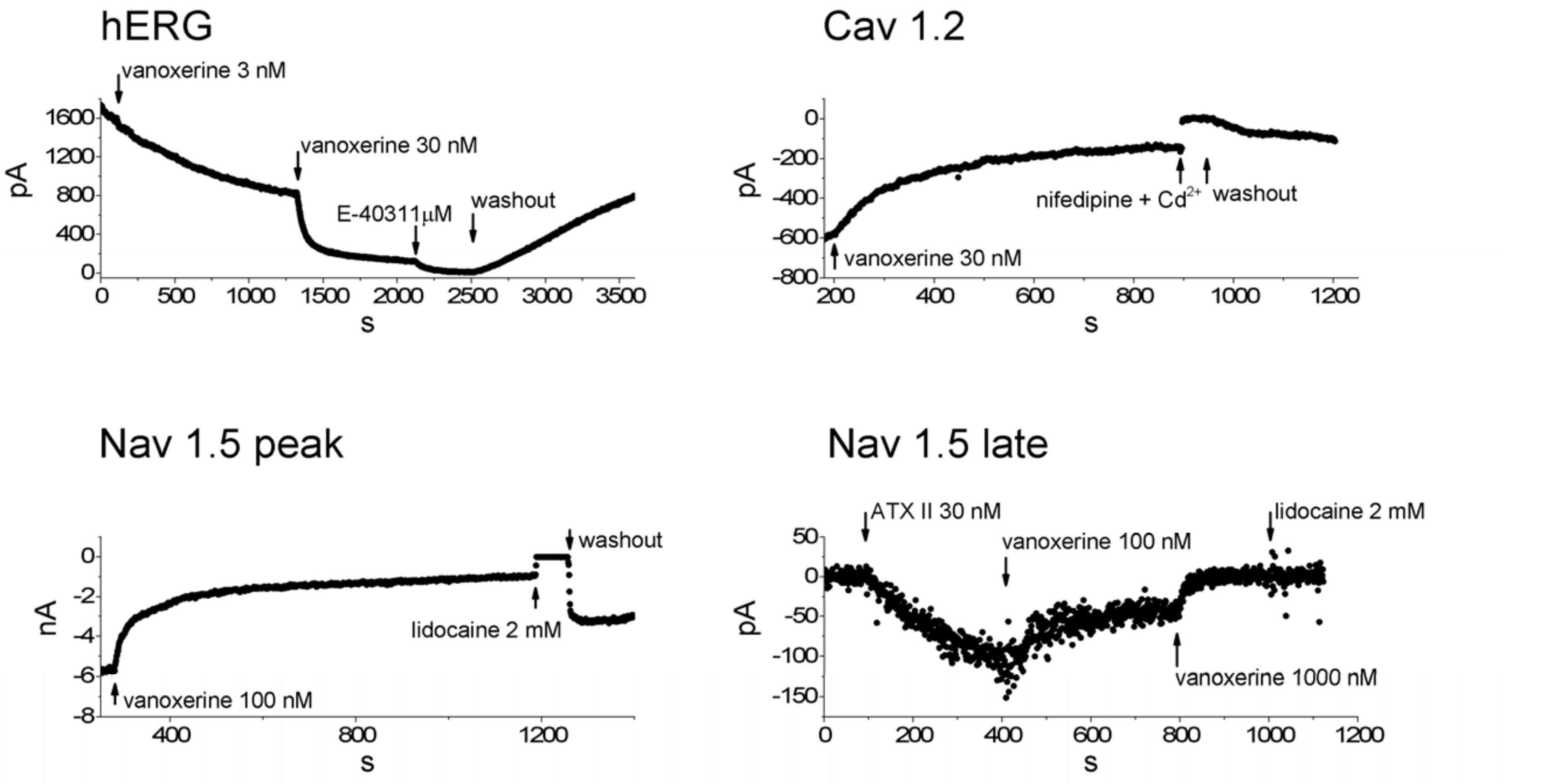

Supplementary Figure 2. Concentration response curves for vanoxerine, bepridil, dofetilide and verapamil
Symbols indicate the mean ± sem and the number of associated experiments. The data was obtained using step-ramps except for hNav1.5 late currents in verapamil where the voltage protocol consisted of a 200 ms pulse to -120 mM, a 5 ms pulse to +50 mV followed by a 400 ms pulse to -30 mV.

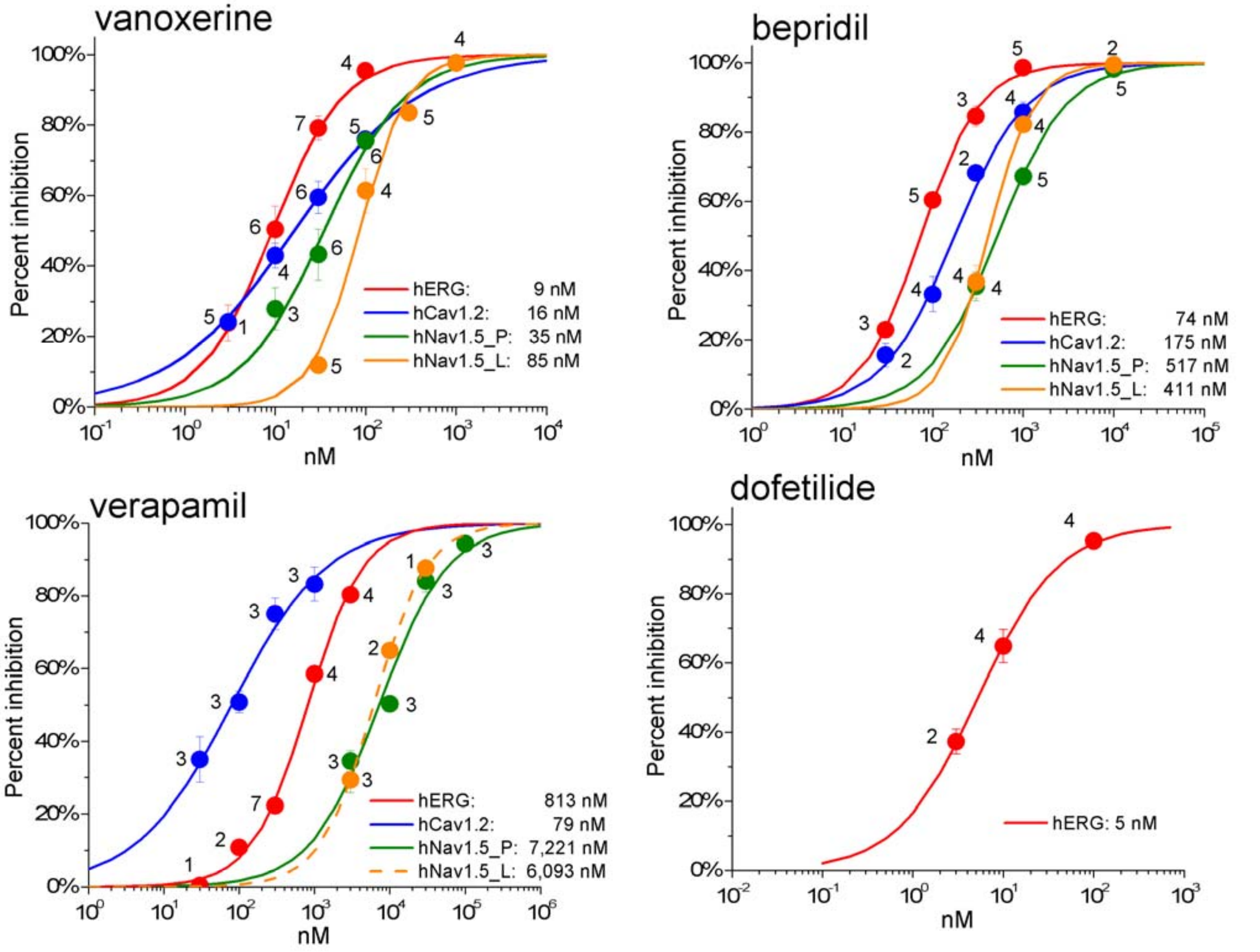

Supplementary

,

Figure 3.
Time course of the changes in membrane potential (top panel), APD30, APD60 and APD90 (black, blue red respectively bottom panel) after exposure to vanoxerine (a), bepridil (b), verapamil (c) and dofetilide (d). Same experiments as those shown in text Figure 3.

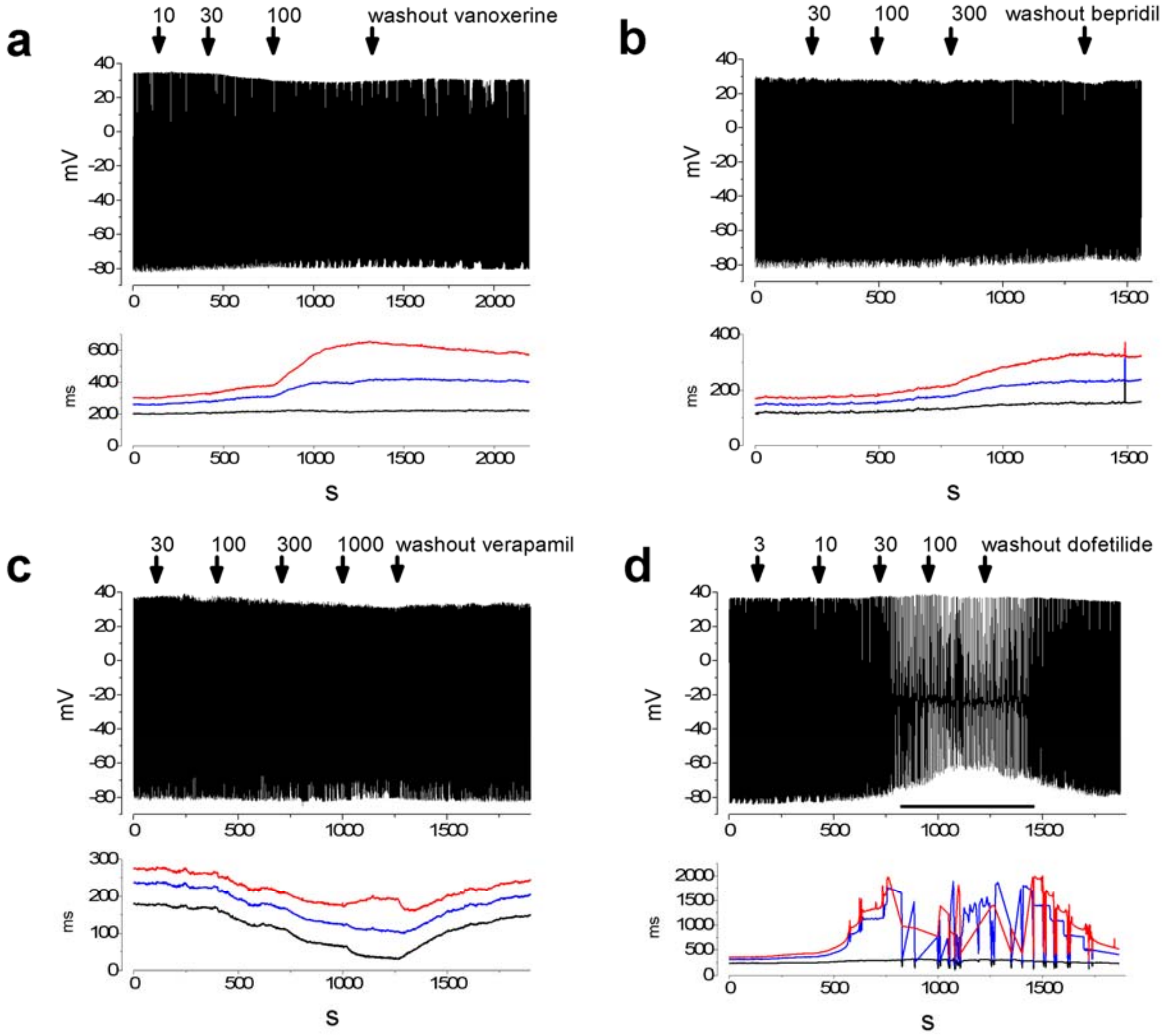

Supplementary Figure 4. Changes in APD90 at steady state as a function of beat period and free drug concentration

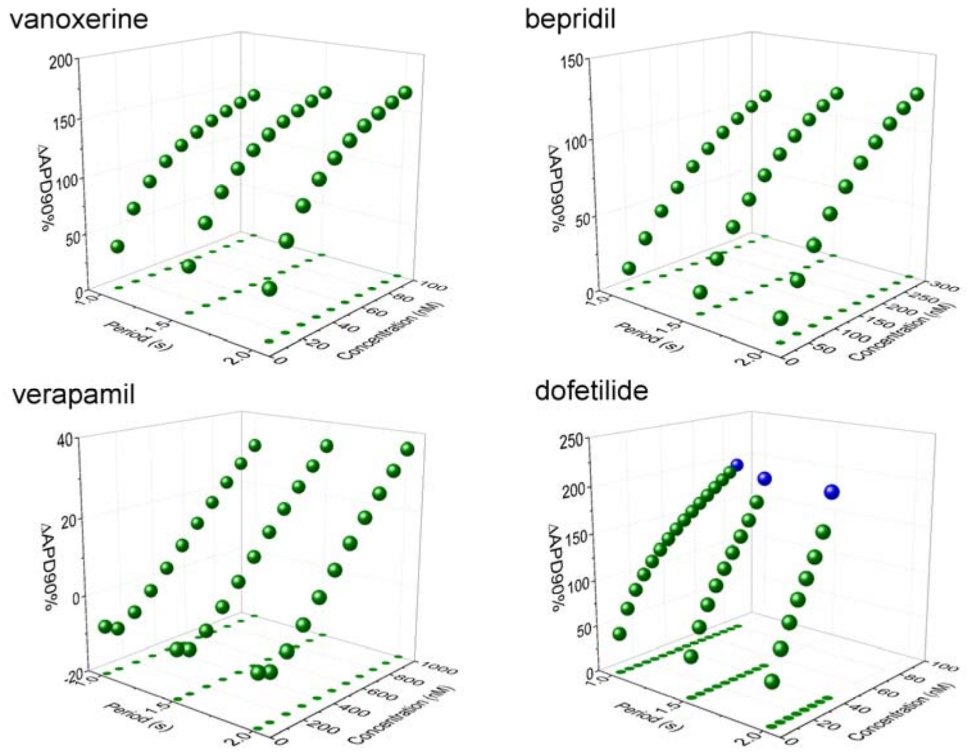

Symbols are APD90 changes in simulations where no triggered activity was observed. The simulations show that vanoxerine prolongs APD in a concentration- dependent manner that is increased at slower frequencies. No arrhythmias were observed. The results show the reverse use-dependent effect of dofetilide since action potential prolongation was greater at slower pacing rates for the same concentration (e.g. 10% larger at 2s compared to 1 s at 10 nM). The projection of the last point of the Period/Concentration plane shows the functional relationship between triggered activity, beat frequency and drug concentration. Triggered activity occurred after the last plotted concentration and ranged from 45 nM (2 second period) to 80 nM (1 second period). Bluesymbols indicate the last AP in the series showing no arrhythmic markers. Verapamil at concentrations lower than 200-300 nM reduced APD90 but at larger concentrations increased APD90. No arrhythmic activity was observed. Bepridil prolonged APD90 but showed no proarrhythmic markers.

Supplementary Figure 5.
Examples of the effects of vanoxerine, bepridil, verapamil and dofetilide on simulated action potentials at 1 Hz. Line colors are consistent with the experiments shown in Figure 3 in the text.

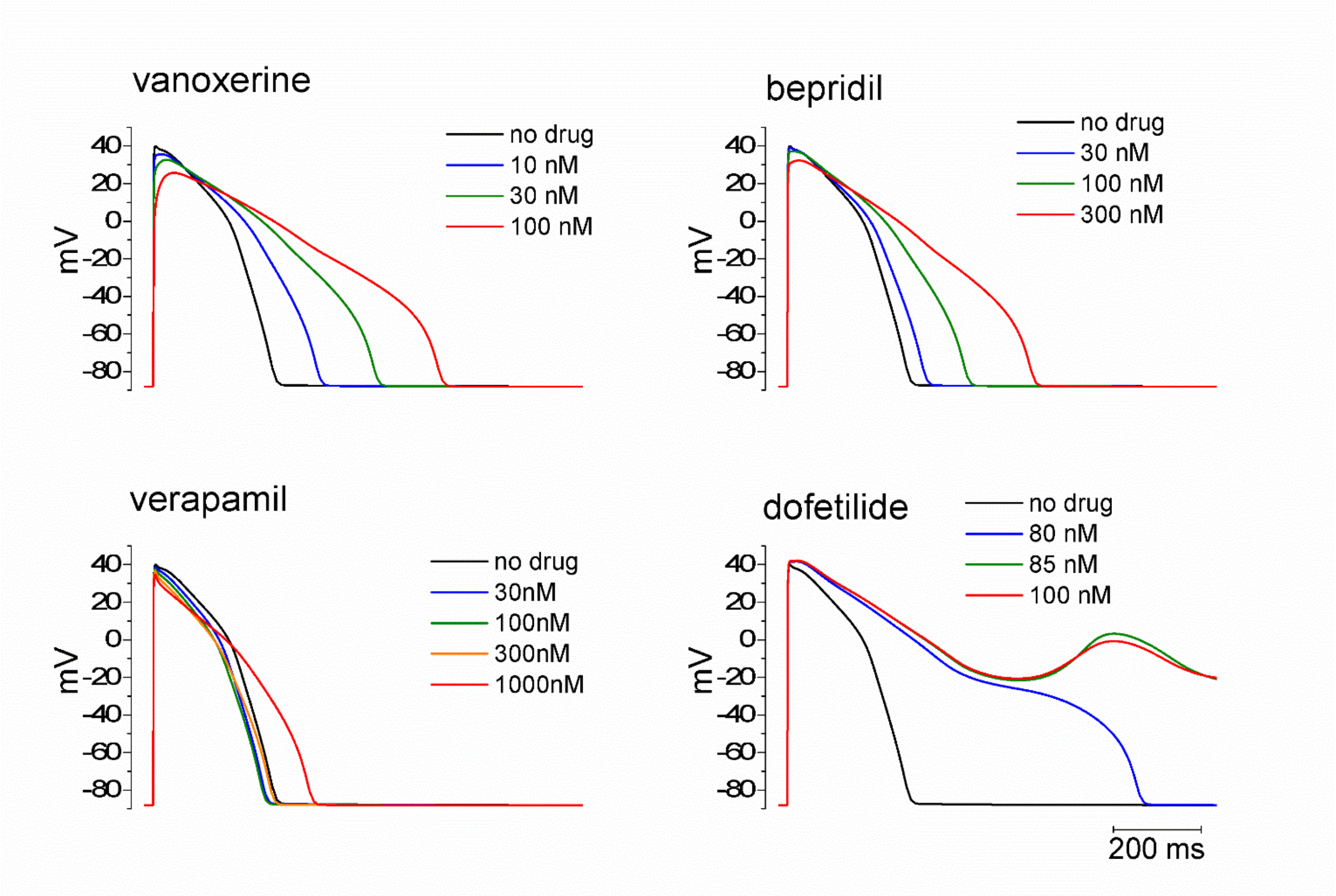

Supplementary Figure 6. Non-specific tissue binding: an explanation of the limited effects of vanoxerine on the arterially-perfused canine wedge preparation.

S 6.1 summarizes the effects of vanoxerine and dofetilide on APD90 of epicardial action potentials recorded in the canine wedge preparation. Data are from Table 4 in Lacerda et al.[2].The percent changes from control were fitted to a logistic function with half maximum effect ($APD90_{half}$) at 206 nM for vanoxerine and 18.2 nM for dofetilide. APD prolongation at $APD90_{half}$ was 3.05% and 11.1% respectively. These values are indicated by vertical lines in the figure. The maximum responses to vanoxerine and dofetilide were 6.1 and 22.6% respectively. Symbols are mean ± sem.

S 6.1

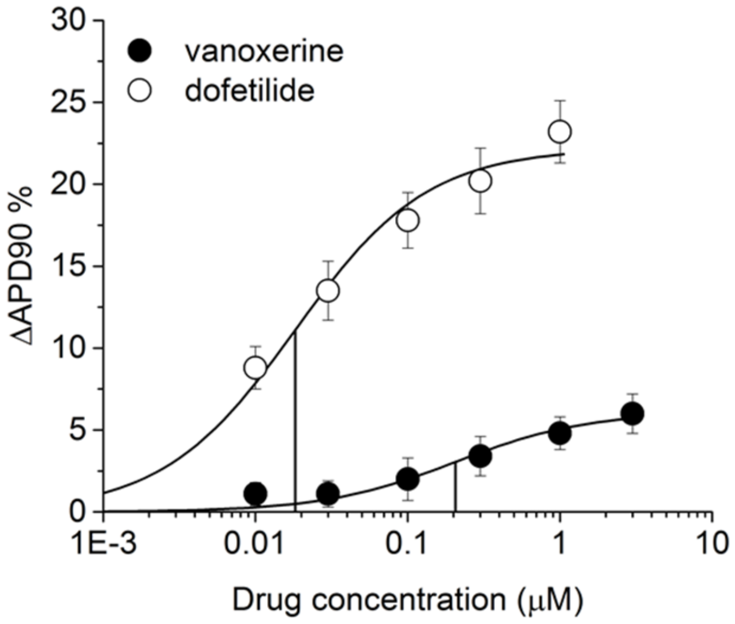

We hypothesized that the limited effects *in situ* are due to tissue binding similar to the binding of the drug to plasma proteins in equilibrium dialysis[1]. To estimate the amount of non-specific tissue binding in the wedge experiments we used *in silico* simulations of the Decker-Rudy model of the canine epicardial action potential[2].

We investigated the effect of stepwise reductions in the vanoxerine concentration from the simulated APD90 shortening at 206 nM free vanoxerine showing a small attenuated AP not at all like a CAP (blue line in S 6.2c) to the experimentally observed prolongation of 3.05 % at $APD90_{half}$. We took as a measure of non-specific (NS) binding the factor that reduces the free concentration from $APD90_{half}$ to a level that produced 3.05% prolongation. Non-specific tissue binding for vanoxerine was calculated at 99.8% consistent with the strong binding measured with the equilibrium dialysis method. Non-specific binding reduced the total drug concentration

from 206 nM to an unbound concentration of 0.5 nM. At that free concentration the shape of the simulated action potential (red line in S 6.2c) is comparable to the action potential recorded in the wedge preparation (S 6.2a).

We used the same approach to assess non-specific binding of dofetilide in the wedge preparation. The model predicts that in the absence of non-specific binding 18.2 nM, the $APD90_{half}$ for dofetilide, should elicit arrhythmic activity (blue line in S 6.2d). However, 89.4% non-specific binding reduced the free concentration from 18.2 nM to 1.94 nM, a concentration that that produced an 11.1% change in APD90. The shape of the simulated action potential is comparable to the shape of the action potential recorded in the wedge preparation (red line in S 6.2d). The measured 89.4% non- specific binding for dofetilide is close to the 60-70% range that is reported (http://dailymed.nlm.nih.gov/dailymed).

S 6.2

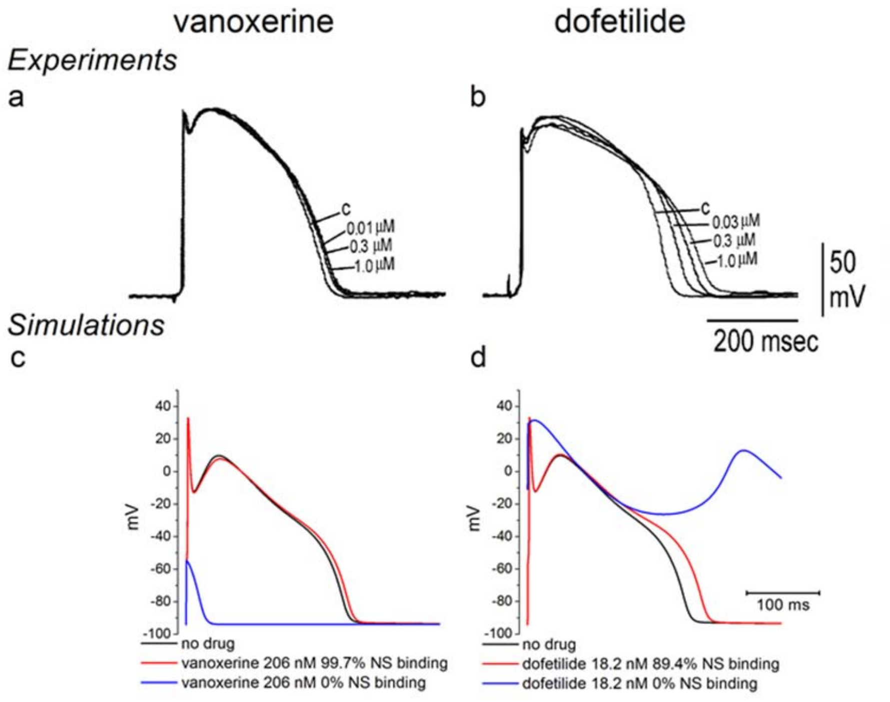

a, **b**) Examples of endocardial action potentials recorded in the presence of vanoxerine and dofetilide in the canine wedge preparation (Data from Lacerda et al., 2010[1]). **c,d**) Simulated action potentials in the presence of vanoxerine and dofetilide under steady state conditions and conductances reduced based on the free drug concentrations and IC50s shown in Table 1 in the text. Black lines: no drug; blue lines: simulation based on $APD90_{half}$ (Note the lack of concordance with the experiments in **a** and **b**); red lines: action potential waveforms calculated assuming 99.8% and 89.4% non-specific binding for vanoxerine and dofetilide respectively. Action potentials were simulated using the Decker-Rudy cardiac epicardial dog model[3]. Simulations used the conductance-block approach where maximal conductances were modified according to the measured IC50s and Hill coefficients (**Table 1)**. Two thousand action potentials were simulated to ensure attainment of steady state.

References

1. Lacerda, A. E., Kuryshev, Y. A., Yan, G. X., Waldo, A. L. & Brown, A. M. Vanoxerine: cellular mechanism of a new antiarrhythmic. *J. Cardiovasc. Electrophysiol*. **21**, 301-310 (2010).

2. Decker, K. F. & Rudy, Y. Ionic mechanisms of electrophysiological heterogeneity and conduction block in the infarct border zone. *Am. J. Physiol. Heart Circ. Physiol*. **299**, H1588-97 (2010).
